# I am happy for us: Neural processing of vicarious joy when winning for parents versus strangers

**DOI:** 10.3758/s13415-020-00839-9

**Published:** 2020-10-14

**Authors:** Philip Brandner, Berna Güroğlu, Eveline A. Crone

**Affiliations:** 1 Erasmus School of Social and Behavioural Sciences, Erasmus University Rotterdam, Rotterdam, The Netherlands; 2 Institute of Psychology, Leiden University, Leiden, The Netherlands

**Keywords:** Vicarious joy, Empathy, Nucleus accumbens, Family, Emotional closeness

## Abstract

This study investigated the neural processes underlying vicarious joy and their dependence on emotional closeness. Prior studies revealed that the nucleus accumbens (NAcc) is a target brain region for processing rewards for self, but the neural mechanisms of processing rewards for others are not yet well understood. A functional magnetic resonance imaging (fMRI) paradigm was employed in young adults (*N =* 30), in combination with a self-report questionnaire on the perceived emotional closeness to the target. We examined the neural correlates of vicarious rewards when winning money for oneself or one of three other targets. To examine family relationships, two of the targets were the mother and father of the participants, and the third target was an unknown stranger. We found an increase in activation in the NAcc when playing for family members compared with a stranger. We further observed a difference in neural activation when winning for the father compared with the mother in an extended network involving the medial prefrontal cortex and precuneus, brain regions involved in mentalizing. These findings were not related to reports of emotional closeness. This new paradigm has considerable value for future research into the fundamental neural processes underlying empathy and vicarious joy.

**Electronic supplementary material:**

The online version of this article (10.3758/s13415-020-00839-9) contains supplementary material, which is available to authorized users.

## Vicarious joy and reward

Vicarious joy is the ability to feel happy about other people’s positive experiences (Batson et al., 1991). In that sense, it differs from compassion in its valence of the shared emotion, focusing on the positive rather than the negative experience of another person (Royzman & Rozin, 2006). Vicarious joy can further be conceptually defined by its focus on the other person’s positive experience rather than one’s own feeling of positivity, which is sometimes defined as *warm glow* (Andreoni, 1990; Batson et al., 1991). In that sense, vicarious joy requires the ability to cognitively grasp someone else’s emotional state, also referred to as mentalizing. Without this key foundational ability, one would just feel the positive emotional contagion of someone else’s happiness, without necessarily understanding why (Schnell, Bluschke, Konradt, & Walter, 2011). Empathy, especially with negative valence, has been the focus of a substantial amount of research in the past decade. For most of these studies, participants are expected to show compassion for someone else’s negative experiences (Batson, 2009; Decety & Ickes, 2009; Preston & De Waal, 2002; Zaki & Ochsner, 2012). Research focusing on vicarious joy, on the other hand, seems to be more elusive.

One controlled way to investigate vicarious joy is by focusing on one specific instance of it: When receiving monetary rewards for others, also referred to as vicarious reward processing.

Reward processes can be investigated with a combination of behavioral measures and neuroimaging techniques. Neuroscientific studies into reward processing have found the striatum, a brain region in the basal ganglia, to be critically involved in these processes (Apicella, Ljungberg, Scarnati, & Schultz, 1991; Apicella, Scarnati, Ljungberg, & Schultz, 1992; Kawagoe, Takikawa, & Hikosaka, 1998). The ventral part of the striatum, the nucleus accumbens (NAcc), specifically, codes for the impact of reward-related stimuli as well as approach behavior in rodents (Robbins & Everitt, 1992). In humans too, the ventral striatum has been found to code for reward processing (Berridge & Kringelbach, 2015; Delgado, 2007; Liu, Hairston, Schrier, & Fan, 2011). Over the past 2 decades, robust evidence has pointed to the ventral striatum as a general-purpose, currency-independent, reward-processing unit responding to various rewards, including monetary rewards, food, and social interactions with others (Delgado, 2007; Knutson, Fong, Adams, Varner, & Hommer, 2001b; Sescousse, Caldú, Segura, & Dreher, 2013).

## Neural mechanisms of vicarious reward processing

The psychological and neural mechanisms of how individuals vicariously share the feeling of other people’s rewards has recently received more attention. It has become evident that sharing others’ positive emotions depends on multiple social factors (Varnum, Shi, Chen, Qiu, & Han, 2013), including the social and emotional closeness we experience to the other person (Fareri, Niznikiewicz, Lee, & Delgado, 2012). These studies show that individuals are more likely to share a positive emotional state with someone else winning a reward if that person is emotionally close to them. It is therefore relevant to take the participants’ emotional and social closeness to the target into account when investigating neural mechanisms of vicarious reward processing (Braams & Crone, 2017).

An important question concerns whether reward processing for oneself, which we observe in the ventral striatum, also extends to others. A recent meta-analysis examined this question by running an activation likelihood estimate analysis on a set of 25 neuroimaging studies. All of these studies investigated some form of vicarious reward processing with the targets of this vicarious context ranging from strangers to friends and family. The results show that the ventral striatum, across the set of 25 studies, was selectively activated for personal reward and not for vicarious reward (Morelli, Sacchet, & Zaki, 2015). Some of these studies, however, found activation for socially close others in the ventral striatum (Braams & Crone, 2017; Fareri et al., 2012; Telzer, Fuligni, Lieberman, & Galván, 2013; Varnum et al., 2013), with the targets being usually a close friend or the mother. One study specifically examined the link between ventral striatum neural activity for vicarious reward situations and the socioemotional distance to the target. The findings suggest activation patterns for a close friend similar to oneself but did not find this same result for a stranger (Braams, Güroğlu, et al., 2014a). In other words, there is tentative evidence to assume we indeed experience a more affective feeling of vicarious joy for close others compared with distant strangers (Mobbs et al., 2009).

## Family relationships and feeling joy for others

This study examined vicarious reward processing for direct family members. The relationship individuals experience with their parents is a foundational building block for social development, emotion regulation (Morris, Silk, Steinberg, Myers, & Robinson, 2007), and empathy (Padilla-Walker & Christensen, 2011). Negative relationships with parents during childhood have long-lasting effects on the mental health of the children (Morgan, Brugha, Fryers, & Stewart-Brown, 2012). Meta-analyses have shown a small effect size for the relationship between parenting and children’s anxiety (McLeod, Wood, & Weisz, 2007b) and depression (McLeod, Weisz, & Wood, 2007a). Intriguingly, an overwhelming majority of research has specifically focused on the mother–child relationship, while mostly relegating fathers to the investigative sideline. We paid deliberate attention to account for the participants’ relationship with both parents in our study.

## The current study

In the current study, we investigated the personal and vicarious neural reward processing by using a false-choice task, comparable to a heads-or-tails gambling task (Braams & Crone, 2017), in a new fMRI design. The reason for using a false-choice paradigm was to dissociate reward processing from other processes that could influence the neural signal for vicarious joy, such as motor learning or reinforcement learning (Tamir & Hughes, 2018). Prior studies reported that these paradigms are better suited than passive paradigms because perceived volition modulates reward-related activity in the striatum (Rao, Korczykowski, Pluta, Hoang, & Detre, 2008; Zink, Pagnoni, Martin-Skurski, Chappelow, & Berns, 2004). This paradigm’s novelty stems from the focus on both the mother and the father, as well as the addition of simultaneous player reward, not just of others, as in previous research. This allows for a more nuanced investigation of vicarious reward, not just for different targets but also in relationship to one’s own personal reward. The parental targets of vicarious reward were baselined against an unknown stranger allowing for nonfamily (outgroup) control conditions. We investigated the neural processing during reward outcomes where each trial of our paradigm referred to a specific combination of rewards for oneself and for one other target individual, who could be the participant’s mother, father, or an unfamiliar stranger (Spaans, Burke, et al., 2018a). We hypothesized that winning for oneself would elicit higher activation from the ventral striatum than would winning for all other targets (Morelli, Knutson, & Zaki, 2018). We expected the relationship with the other person to be associated with differences in neural responses to vicarious rewards. We anticipated the ventral striatum to show no difference in neural response when the stranger received a reward compared with the condition where no one wins any reward. We expected, however, to find stronger ventral striatum activation for rewards for parents than for rewards for strangers (Braams & Crone, 2017). We had no specific expectations for the comparison of mother and father conditions. However, based on prior studies showing that mother–child relationships are often reported to be closer than father–child relationships, we explored vicarious reward processing for mothers and fathers separately (Russell & Saebel, 1997; Solomon, Warin, Lewis, & Langford, 2002).

## Method

### Participants

For this study, 32 adult participants were recruited (17 females; *M*_age_ = 22.5 years, *SD* = 1.5 years). Based on previous literature (Braams, Güroğlu, et al., 2014a; Spaans, Peters, & Crone, 2018b) reporting medium effect sizes in similar experiments (*f* = 0.30) we used G*Power (Version 3) to calculate a sample sizes based on alpha error probability of 0.05 (and hence a statistical power of 0.95) for a repeated-measures analysis of variance (ANOVA). This was the primary statistical tool for our region of interest (ROI) main confirmatory hypothesis. The power analysis resulted in a suggested sample size of 31. We were able to recruit 32 participants for this study, and included 30 in the final analysis. The data for this study were collected as part of a larger longitudinal fMRI study on prosocial behavior, funded by the European Research Commission. The child or self yield (COSY) task was one of two different fMRI tasks that were performed by the participants in the study.

The majority of the participants were undergraduate university students, and Caucasian. The authors are aware that not all families are “traditional,” in the sense of not only having a male and a female parent but also in the sense of having two parents in the household. For paradigm simplicity and focus on a homogenous sample, we decided to only include participants with both a mother and a father. Participants were screened for MRI contra-indications and for a history of neurological and/or psychiatric disorders. All anatomical MRI scans were reviewed by a radiologist; no anomalous findings were reported. All participants gave informed consent before the start of the study.

### Procedure

Upon arriving at the scanning session, participants received general instructions before completing a practice run of the experimental paradigm to be played in the MRI setting. After a scan session of about 1 hour, the participants received a short questionnaire inquiring about their experiences during the scanning. The total duration of the data collection session per participant was around 2 hours. Two participants were excluded due to excessive head movement (translational spikes above 3 mm), so the reported results are based on 30 participants (16 female; *M*_age_ = 22.8 years, *SD* = 1.5 years). Participants received financial compensation of 40 euro, plus an additional amount between 3.30 euro and 6.50 euro, depending on their choices and outcomes in the tasks they performed. Out of this additional reward, 1 euro was specific to the vicarious reward (COSY) task. The study and all procedures were approved by the medical ethical committee of the Leiden University Medical Center (Protocol Number NL62878.058.17).

### Materials

#### COSY fMRI task

**Fig. 1:**
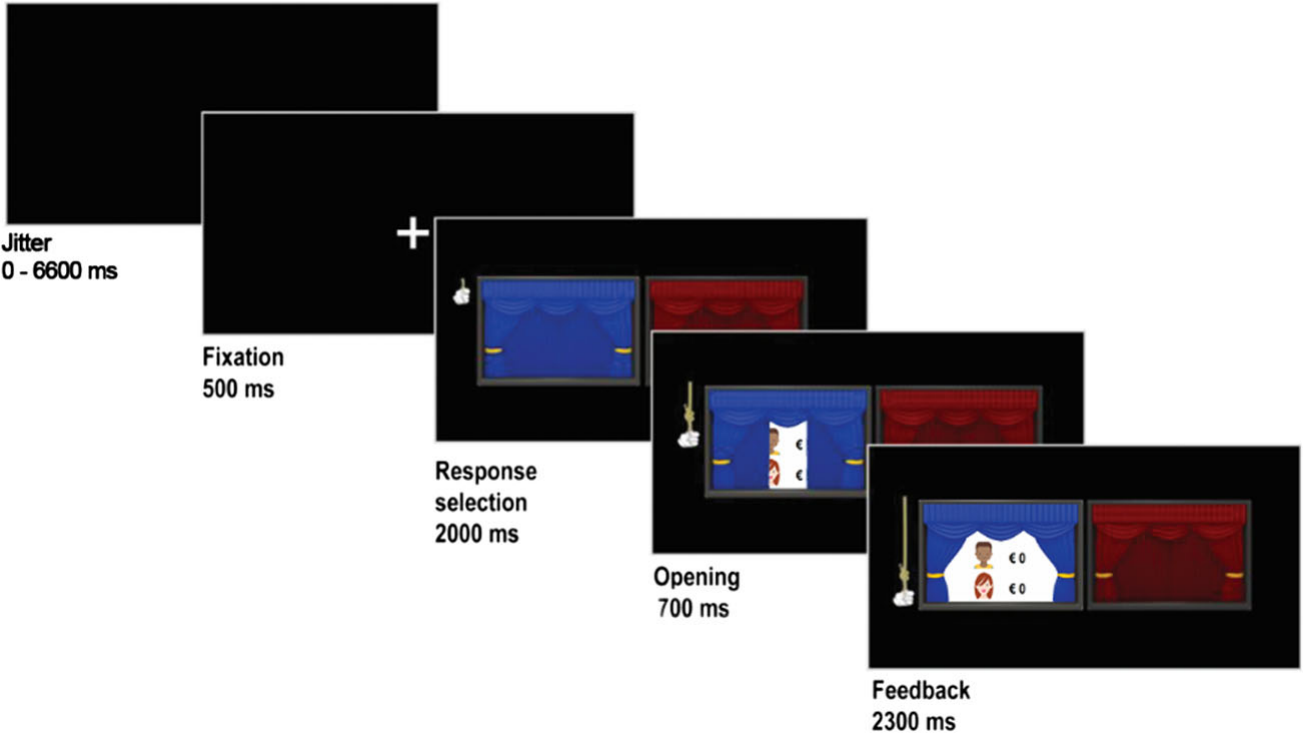
Design of the vicarious reward task. Starting with a randomized jitter (0–6,600 ms) each trial is followed by a fixation cross and the stimulus onset showing two closed curtains. The participant has the option to choose between the left and right curtain. The chosen side opens with an animation followed by a feedback presentation showing the reward outcomes for the participant and one of the three targets. The probabilities for all monetary outcomes were identical for all participants due to the false-choice nature of the paradigm. The player’s choices between left and right curtain did not influence the outcome

In the current study, an adapted version of the COSY task was used to examine the neural underpinnings of vicarious reward processes (Spaans, Peters, et al., 2018b). During the introductory session before scanning, the participants were instructed that they would play a game with their mother, their father, and a stranger (i.e., another participant of the study, as explained at the start of the scan day). No additional information regarding the stranger’s gender, race or socioeconomic background was given. Parents were not present during the experiment but participants were aware that they would receive a real monetary reward for themselves and their parents, depending on task outcomes. At the beginning of each trial, a jitter (black screen) was used for 15% of the entire task time, ranging from 0 to6.6 seconds, followed by a fixation cross for 500 ms, independent of the jitter. The stimulus presentation started with a screen presenting two curtains (one red, one blue) where participants were asked to choose one with a button press (they had 2 seconds to make this decision). They always only had the option to choose the left or the right curtain, with the outcome being random, there was no way for the participants to influence the reward outcomes (see also Braams & Crone, 2017, for a similar heads-or-tails gambling format). Once they pressed a button, a hand icon holding onto a rope to open the curtains appeared next to the curtain the participant chose. After 2 seconds, the hand icon pulled down the rope and revealed the monetary rewards behind the curtain of choice (the opening animation lasted for 700 ms and was visualized using 15 distinct images of the curtains). The reward outcome was unknown to the participant during the response selection. Rewards were always displayed for the participant at the top of the screen, with the outcome of one of the three targets visualized underneath. This feedback was presented for 2.3 seconds, which marked the end of each trial. The probabilities for all monetary outcomes were identical for all participants due to the false-choice nature of the paradigm. The player’s choices between the left and right curtain did not influence the outcomes provided in the task. All participants received an equal number of all possible outcomes of the task (see Fig. 1).

If the participant did not press a button within the 2 s where he or she could choose one of the two curtains, an on-screen text informed that he or she was “too late” for the duration of 1 second, and was followed by the next trial. The task consisted of four conditions: (i) “NoWin” condition, entailing an outcome of zero euros for both players; (ii) “BothWin” condition, entailing 1 euro for both players; (iii) “OtherWin” condition, entailing 2 euros for the target and nothing for the participant; and (iv) “SelfWin” condition, entailing 2 euros for the participant and nothing for the target. There were 15 trials of each of the four conditions and for each of the three players, resulting in a total of 180 trials. The trials were randomized by both the identity of the three target partners as well as the monetary outcome conditions. The paradigm design was optimized for efficiency using optseq2 (Dale, 1999).

Half of the participants (*N* = 16) were presented with a collection of flat-icon avatars, where they could choose the icons that would represent themselves, their mother, and their father during the task. The rest of the participants (*N* = 14 after exclusion of participants) were shown the target stimuli in the form of text (e.g., monetary reward for mother was identified by the word “mother” next to it). This was done to provide the participants with meaningful visual representations of themselves and their family members. We did not have any strong a priori hypothesis and we analyzed neural differences between the text and icon group participants. The findings did not differ across participants who saw an icon or text as labels for the target; the results are thus presented here by averaging across all participants.

### Behavioral measures

After the MRI session, the participants were asked several questions regarding the COSY task they had just played and the targets involved.

#### Pleasure from winning

The participants were first asked to indicate how much they liked winning money for themselves and for each of the three targets. All answers were given on a scale ranging from 1 (*did not like it at all*) to 7 (*liked it a lot*).

#### Closeness

To assess how close the participants felt towards the other target we used the Inclusion of Other in Self (IOS) scale (Le, Moss, & Mashek, 2007), which is a measure of self–other inclusion and relationship closeness. The participants are asked to indicate how close they feel to the other person based on a 7-point scale that uses two Venn diagrams that are placed next to one another, ranging from no overlap (1 = *no inclusion of other in self*) to almost complete overlap (7 = *almost completely overlapping other and self*). Participants were asked to fill out the IOS separately for their mother, their father, and the stranger that they played the COSY task with.

### MRI acquisition

Participants were scanned on a Philips Achieva 3.0 Tesla scanner using a 32-channel head coil. Following the localizer scan, first a T1-weighted structural scan was recorded (isotropic voxel size 1.1 mm^3^, RT = 7.9 ms, TE = 3.5 ms, flip angle = 8 degrees, FOV = 250 mm, duration = 04:12 s) using a 3DT1 image sequence. Next, a T2* functional scan was performed (voxel size = 2.75 mm × 2.84 mm × 2.75 mm, RT = 2.2 s, TE = 30 ms, flip angle = 80 degrees, slice thickness, echo planar imaging [EPI], volumes = 3 × 188, number of slices = 38, FOV = 220 mm, duration = 3 × 07:09 s). Functional scans consisted of three runs, with 188 volumes each, and each run lasting 6 minutes; we discarded the first two scans to allow for stabilization of the signal. Participants were instructed to lie still in the scanner and were constantly monitored through a camera system. Furthermore, head movements were restricted by using foam triangles to fill available empty space between the participant’s head and the head coil.

### Preprocessing

Neuroimaging data were preprocessed and analyzed using SPM8 (Wellcome Trust Centre for Neuroimaging, London) and internal MATLAB scripts. For preprocessing, we corrected all functional scans for slice timing and excessive head motion (6 parameters). Following the coregistration of the T2* with the structural scan, we resampled all volumes to the resolution of 3 mm^3^. Normalization to an anatomical atlas was based on MNI305 (Cocosco et al., 1997). Finally, we used an isotropic Gaussian kernel (6 mm FWHM) to spatially smooth the data.

### FMRI analysis

We modeled the fMRI time series with the hemodynamic response function (HRF) convolution and with the outcome timings of each condition. This allowed us to create contrasts to be used during the first-level analysis. We modeled the first moment of reward outcome presentation (Image 7 within the opening animation) as a null duration event for each of the four outcome conditions: NoWin, BothWin, SelfWin, and OtherWin, for each of the three targets, resulting in 12 conditions in total. All of these events were time locked with a zero duration to the exact moment that participants were able to see the first image of the monetary reward (i.e., the seventh frame of the curtain-opening animation; see Fig. 1, above). Trials without a response or with a late response were coded as invalid and excluded from further analysis. A general linear model (GLM) was created using all 12 conditions, along with motion regressors and a high-pass filter of 120 Hz. The (least square) parameter estimates (beta weights) of the best fitting canonical HRF for each condition were used in pair-wise contrasts. These contrasts were then used in the random-effects group analysis. Contrast appropriate false discovery rate (FDR) cluster level thresholds were chosen for all whole-brain analyses (see figures for details).

### Region of interest (ROI) selection

To investigate the neural activation patterns of vicarious rewards for parents and strangers, we performed NAcc) ROI analysis using the MarsBaR toolbox in SPM8 (Brett, Anton, Valabregue, & Poline, 2002). This bilateral region in the ventral striatum was chosen for its robust role in reward and prediction processing. ROI selection was based on a predefined anatomical ROI of the left and right NAcc as extracted from the Harvard–Oxford subcortical atlas and thresholded at 40% (for details, see Braams, Van Duijvenvoorde, Peper, & Crone, 2015). The ROI mask consists of 28 voxels for the left NAcc (coordinates left: *x* = −9.57, *y* = 11.70, *z* = −7.10) and 26 voxels for the right NAcc (coordinates right: *x* = 9.45, *y* = 12.60, *z* = −6.69). Following the ROI selection, we extracted parameter estimates for the analysis. None of the results showed differences between the left and right NAcc; therefore, all the analyses were performed by collapsing across both hemispheres (see Supplementary Material). To be able to investigate the neural activation for winning for self and others, we used the NoWin condition as the baseline in the task. In examining the neural activation for winning for the self, we examined the SelfWin–NoWin contrast; for neural activation when winning for the target, we examined the OtherWin–NoWin contrast; and finally, for the BothWin condition, we examined the BothWin–NoWin contrast.

All reported T-map contrasts have been uploaded to NeuroVault.org (Gorgolewski et al., 2015): https://neurovault.org/collections/UNRMPFBJ/.

## Results

### Behavioral results

#### Pleasure from winning

**Fig. 2:**
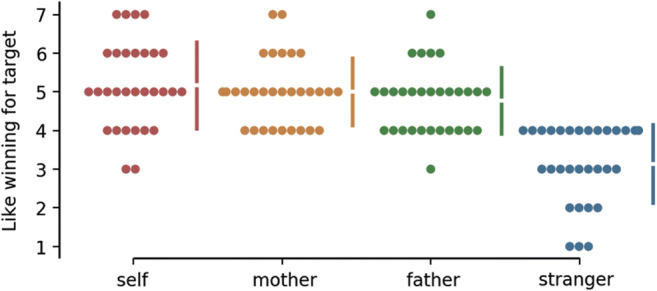
Results for like winning for self, mother, father and stranger are shown in the above Cumming estimation plot. Each data point is depicted as a dot. Each 95% confidence interval is indicated by the ends of the vertical error bars

To validate task manipulations independent of neural activity, a behavioral analysis was conducted first. A repeated-measures ANOVA was conducted to examine the role of the target (four levels: self, mother, father, stranger) on how much participants enjoyed winning; a main effect of the target was found, *F*(3, 116) = 28.1, *p* < .001 (see Fig. 2). Post hoc Tukey tests confirmed our expectation that pleasure of winning for a stranger (*M* = 3.1) was significantly lower compared with self (*M* = 5.1, *p* < .001), mother (*M* = 5.0, *p* < .001), and father (*M* = 4.8, *p* < .001), there was no difference between pleasure reported for winning for the self, mother, or father.

#### Closeness

**Fig. 3:**
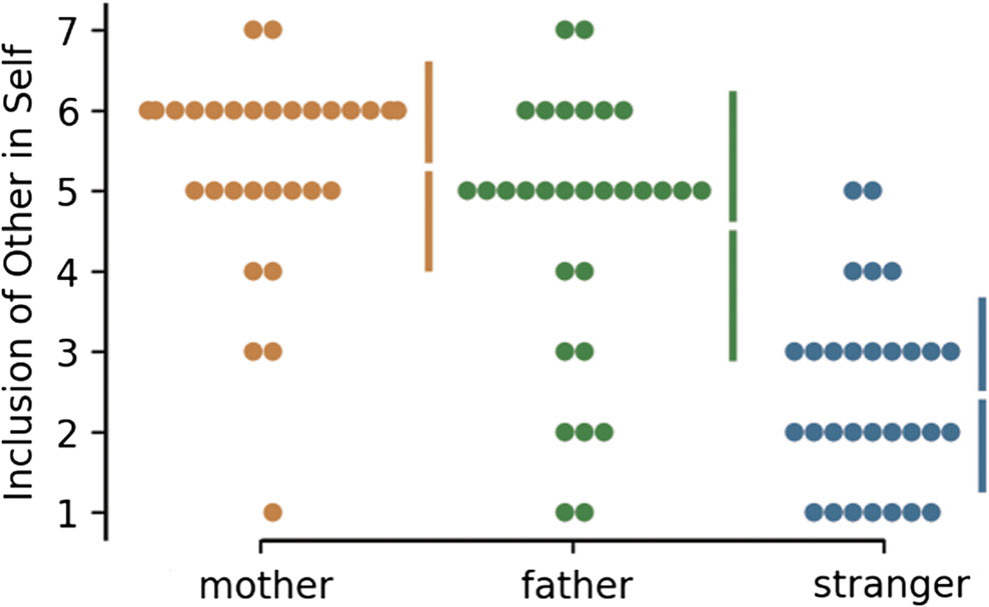
Results for mother, father and stranger conditions for Inclusion of Other in Self (IOS) are shown in the above Cumming estimation plot. Each data point is depicted as a dot. Each 95% confidence interval is indicated by the ends of the vertical error bars

A repeated-measures ANOVA examining how close the participants felt for the other three players (i.e., mother, father, stranger) based on the IOS revealed a significant difference in closeness towards the targets, *F*(2, 58) = 67.90, *p* < .001 (see Fig. 3). A post hoc Tukey test revealed that perceived closeness to a stranger (*M* = 2.5) was significantly lower compared with mother, *F*(1, 29) = 102, *M* = 5.3, *p* < .001, and father, *F*(1, 29) = 62.3, *M* = 4.6, *p* < .001. Closeness felt towards the mother was significantly higher than closeness reported for the father, *F*(1, 29) = 5.20, *p* = .03. The reported closeness to mother was positively correlated with the closeness reported for the father (*r* = .38, *p* = .038). There was no correlation found between the closeness reported to a stranger and either of the parents. No differences between male and female participants were found in any of the behavioral results.

**Fig. 4:**
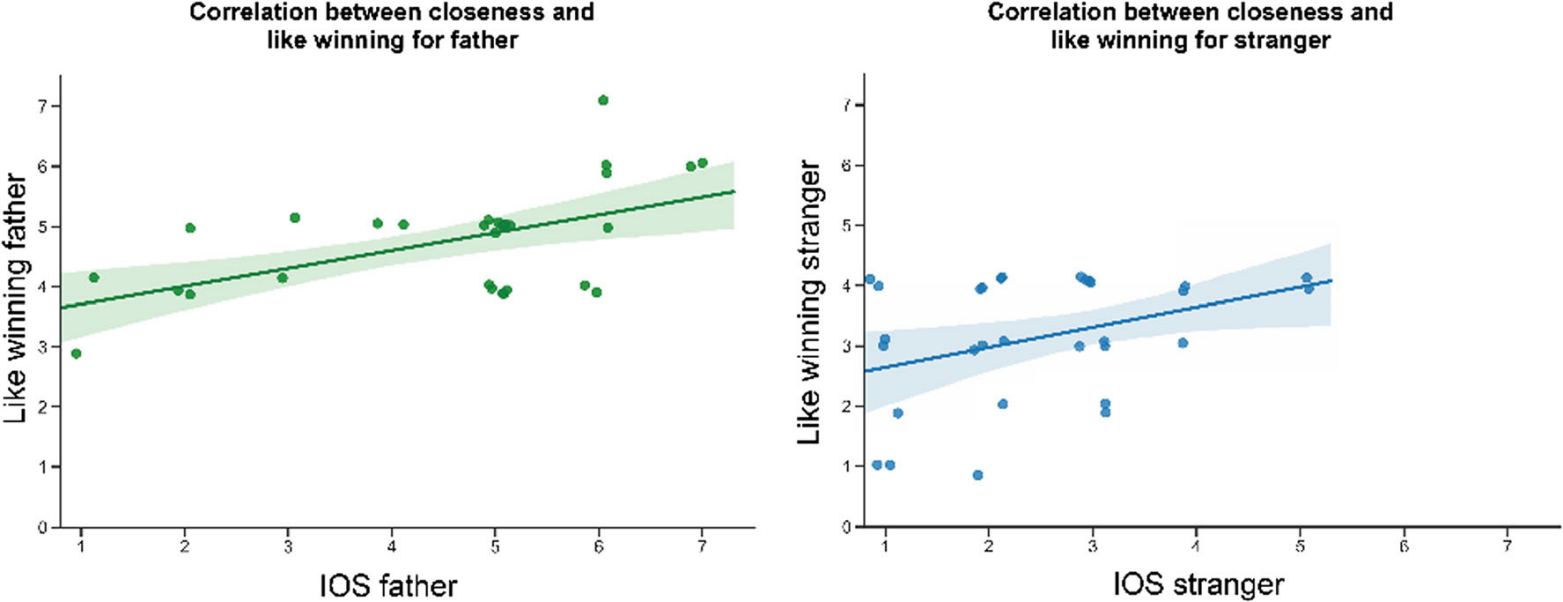
Scatterplots between Inclusion of Other in Self (IOS) and how much the participants liked winning for the target. Left: for father (green). Right: For stranger (blue). Linear regression lines are plotted with a 95% confidence interval around it (green and blue shading). Due to many similar responses on the two Likert scales, a 15% jitter was applied to both axes to allow for better visibility of all data points. The correlation for mothers was not significant and is therefore not displayed. (Color figure online)

**Fig. 5:**
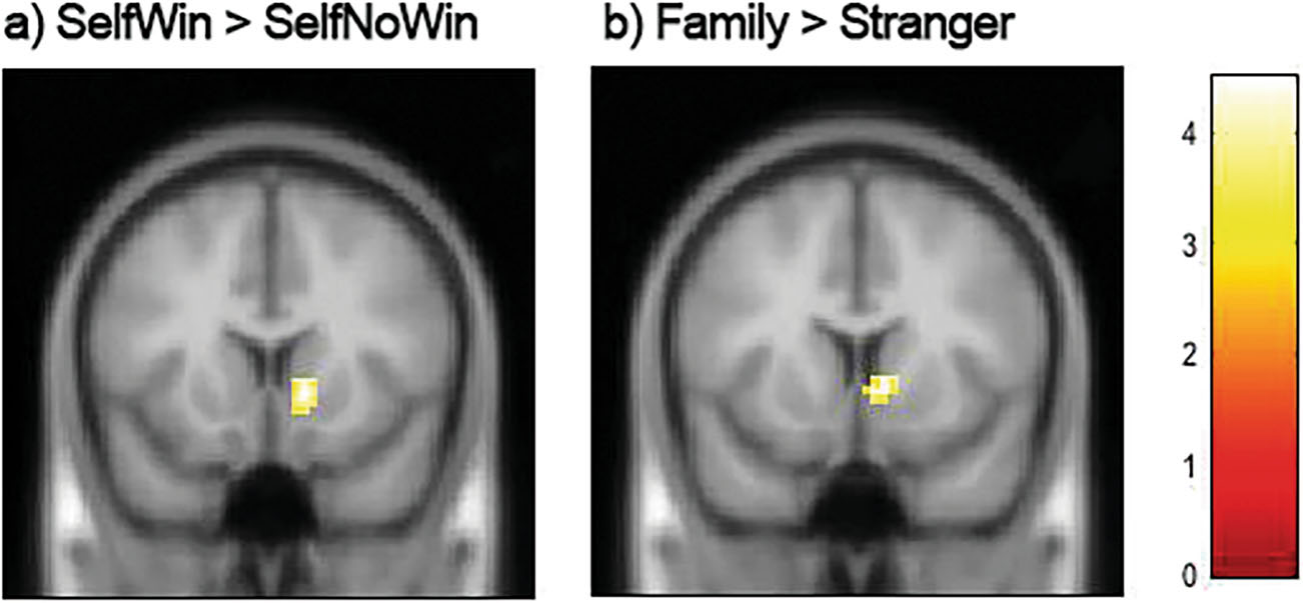
Whole-brain analysis results for two different contrasts. a Right nucleus accumbens (NAcc) activation for SelfWin > Self No-Win independent of outcome for target (primary voxel threshold *p* < .001, FDR cluster corrected = 56). b Right NAcc activation for the contrast Mother+Father > Stranger, across all outcome conditions (primary voxel threshold *p* < .001, FDR cluster corrected = 30)

**Fig. 6:**
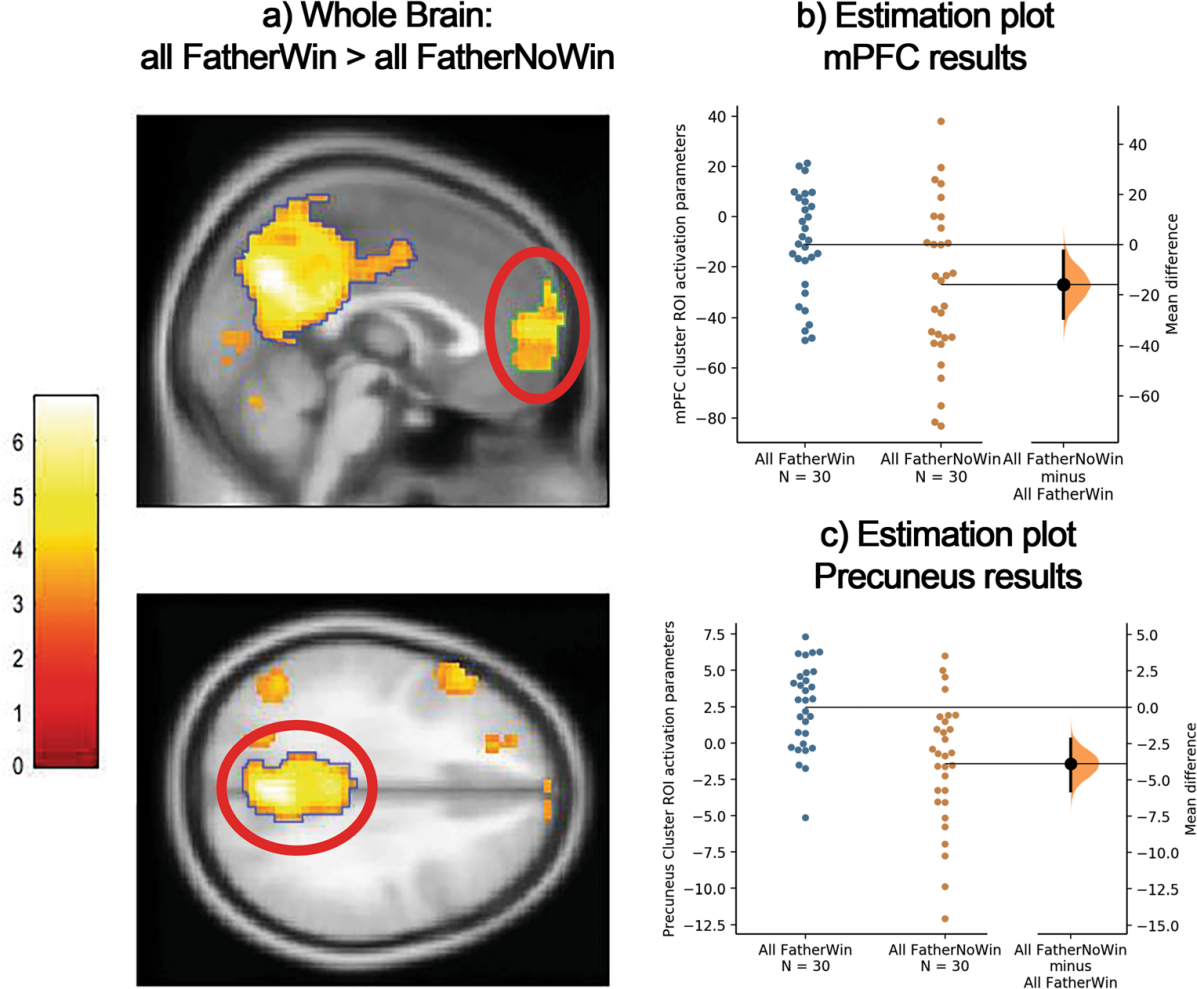
**a** Whole-brain activation pattern for the contrast All FatherWin > All FatherNoWin. Exhibiting activation in the parietal midline (blue outline) as well as the medial prefrontal cortex (mPFC, green outline). Primary voxel threshold *p* = .001, FDR cluster corrected = 77. **b** The mean difference between the two contrasts is shown in the above Gardner–Altman estimation plot for the mPFC ROI results. The two contrasts results are derived from averaging the two conditions that compose them: All FatherWin (FatherWin + BothWin) and All FatherNoWin (SelfWin + NoWin). **c** To get a more precise measurement for this parietal midline cluster midline a stronger FWE, *p* < .05, a voxel-level correction was applied and a functional ROI for the now clearly outlined precuneus was extracted (blue area). The mean difference between the two contrasts is shown in the above Gardner–Altman estimation plot for the precuneus ROI results. The two contrasts results are derived from averaging the two conditions that compose them: All FatherWin (FatherWin + BothWin) and All FatherNoWin (SelfWin + NoWin.Both groups are plotted on the left axes: The mean difference is plotted on a floating axes on the right as a bootstrap (5,000, with replacement) sampling distribution. The mean difference is depicted as a black dot; the 95% confidence interval is indicated by the ends of the vertical error bar

We found positive correlations between IOS scores and like-winning reports for father (*r* = .57, *p* = .001) and stranger (*r* = .39, *p* = .035; see Figs. 4, 5, and 6). There was no correlation for the IOS scores of the mother and like winning reports. We further found a negative correlation between IOS for the stranger and like-winning reports for self (*r* = −.49, *p* = .006) and like-winning reports for mother (*r* = −.37, *p* = .04), showing that less closeness to strangers was associated with more pleasure when gaining for self and for mothers. The above-mentioned *p* values for our correlation analysis are uncorrected. Due to the exploratory nature of this analysis, the decision was made to report all results at an uncorrected threshold of *p* < .05. The correlations were no longer significant when multiple comparison correction was applied.

### FMRI results

#### ROI analysis NAcc

ROI analyses of the NAcc were performed to test specific activity patterns in the a priori hypothesized vicarious reward region. For this analysis, BothNoWin was selected as a baseline area, and all other conditions (SelfWin, BothWin, OtherWin) were presented relative to this baseline condition.

**Fig. 7:**
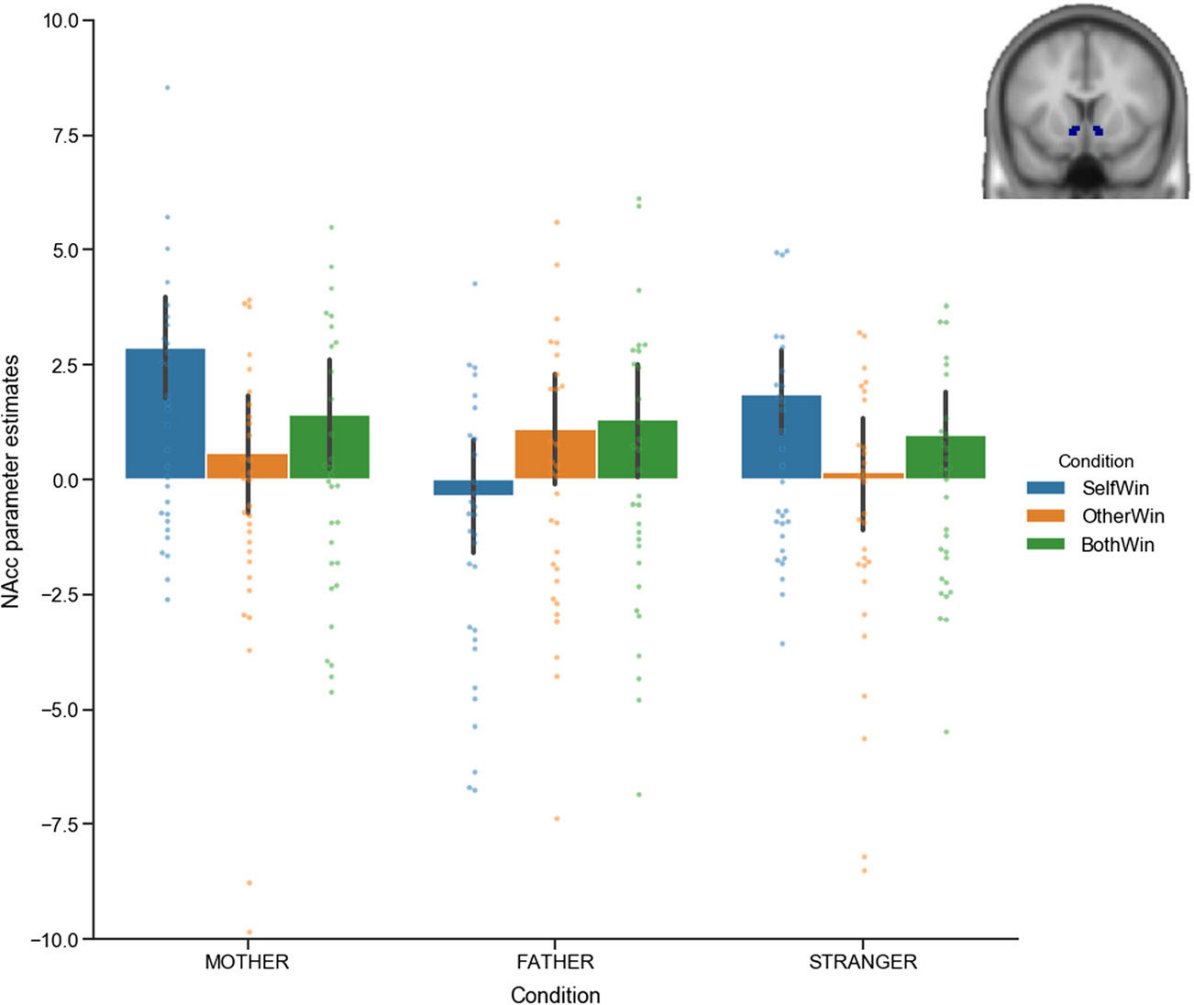
Nucleus accumbens ROI results across all three targets for the three conditions (baselined against NoWin). Both for targets Mother and Stranger the SelfWin condition shows the highest activation, significantly above the baseline of NoWin, with BothWin as second highest and OtherWin as lowest. Structural ROI for bilateral NAcc (blue areas) shown on the top right. Error bars represent 95% confidence interval of the mean (equal to 2× the standard error of the mean, allowing for an easier interpretation of the group comparison due to the focus on the mean difference between groups). Individual datapoints are shown on top of the bar graph

The Target × Condition (3 × 3) repeated-measures ANOVA resulted in, albeit no main effects, a significant Target × Condition interaction, *F*(4, 116) = 6.77, *p* < .001. Follow-up ANOVAs for each condition separately revealed no significant target differences for BothWin, *F*(2, 58) = .16, *p* = .85, or for OtherWin, *F*(2, 58) = .52, *p* = .60. However, the ANOVA for SelfWin resulted in a main effect of target, *F*(2, 58) = 9.29, *p* < .001. Target comparisons revealed that NAcc activity for SelfWin in the father condition was significantly lower than for SelfWin in the mother condition (*p* < .001) and SelfWin in the stranger condition (*p* = .011; see Fig. 7). Mother and stranger did not differ significantly from each other (*p* = .21).

Notable, the outcome SelfWin in the father condition did not differ from the baseline of BothNoWin, suggesting that when playing for self and father, winning for oneself does not result in elevated NAcc activation. In summary, the NAcc ROI findings align with the results of the whole-brain analysis, revealing a difference in activation patterns that are specific to the father and diverge from the mother condition. For additional time series results from the NAcc, see Fig. S5 in the Supplementary Material.

#### Whole-brain analysis

The whole-brain analyses focused on the effects of winning for self and winning for others. The analyses are separated in confirmatory and exploratory analyses. The whole-brain analysis was performed to confirm inclusion of our ROI of interest in our whole-brain findings. Moreover, this also allowed us to examine whether other regions that were not previously considered were also involved in vicarious reward processing using our paradigm, which could potentially be informative for future research.

- ***Winning for self and playing for family.*** To test our confirmatory hypothesis of whether winning versus not winning for self would lead to the expected ventral striatum activity we conducted a whole-brain analysis. All win conditions for self were contrasted to all no-win conditions for self regardless of outcomes for the other targets. This was achieved using a 3 (target) × 4 (all conditions) ANOVA within SPM. This analysis resulted in one cluster of activation in the right hemispheric ventral striatum activation (see Fig. 6a; FDR cluster threshold = 56). The reversed contrast did not result in any significant activation. We further tested our second confirmatory hypothesis testing whether playing for mother or father (across all conditions) compared with the stranger conditions led to ventral striatum activity using the same repeated-measures ANOVA. Indeed, this contrast resulted in one cluster of activation in the right ventral striatum (see Fig. 5b, FDR cluster threshold = 30). We, again, tested for the opposite contrast in this case: stranger > family. No significant activation was found. Finally, we did not find a main effect for either outcome or target; we also did not observe an Outcome × Target interaction effect.
- ***Winning for others****.* Next, we tested neural activity when winning for others. First collapsed across all targets, we contrasted all winning conditions for the other targets (i.e., OtherWin & BothWin conditions) with all no-win conditions for the targets, regardless of outcomes for self (i.e., SelfWin + NoWin). In other words, the two conditions where the other won something (which are BothWin and OtherWin) were contrasted with the condition in which the other did not win something (SelfWin and NoWin). This analysis revealed a cluster of activation in the parietal midline area/precuneus and left midfrontal gyrus (FDR cluster threshold = 61; for details, see Fig. S1 in the Supplementary Material).

We then tested whether this pattern was visible for all targets or for specific partners. For this purpose, we ran these analyses for each of the three targets (mother, father, stranger) separately. We found no activation for the contrast OtherWin > OtherNoWin for mother and stranger (using a voxel threshold of *p* < .001 and a cluster FDR threshold of *p* < .05). For father, the FatherWin > FatherNoWin was associated with activation in five clusters: a larger parietal midline area/precuneus, left inferior prefrontal cortex, left precentral gyrus, right caudate and the mPFC (see Fig. 6, primary voxel threshold *p* = .001, FDR cluster threshold = 77; see also Table 1 in the Supplementary Material). See Fig. 6b for visualization of this effect. Thus, the activation that was observed for winning for others was specific to the father target Table 1.

**Table 1:** Coordinates for whole-brain activation for all contrasts

Contrast	Region (AAL)	T	K (cluster size)	*x*	*y*	*z*
SelfWin > SelfNoWin	Caudate_R	4.31	56	12	14	−2
Family > Stranger	Caudate_R	4.50	33	9	11	−2
	Cuneus_R	3.85	31	3	−61	22
All FatherWin > All FatherNoWin	Precuneus	6.81	3841	0	−64	31
	Frontal_Sup_Medial	5.33	423	−3	68	10
	Frontal_Inf_Tri_L	4.57	212	−57	23	25
	Precentral_L	4.24	268	−33	5	49
	Caudate_R	3.94	78	9	8	1
All OtherWin > All OtherNoWin	Precuneus	4.91	316	0	−61	31
	Parietal_Sup_L	4.31	161	−30	−64	52
	Frontal_Mid_L	4.26	61	−30	8	61
	Frontal_Mid_L	4.24	75	−51	23	51

*Note.* Contrasts: SelfWin > SelfNoWin (FDRc = 56), Family > Stranger (FDRc = 30), and FatherWin > FatherNoWin. FDR cluster corrected (FDRc = 77 voxel, initial voxel threshold *p* = .001 for all contrasts). The Automated Anatomical Labeling (AAL) Atlas was used for labeling. MNI coordinates are shown for peak voxel within each cluster. All reported statistical group T-maps have been uploaded to Neurovault.org (https://neurovault.org/collections/UNRMPFBJ/)

### Brain–behavior relations

To investigate the relation between the NAcc activation when winning for self and others and our behavioral measures, we first examined the correlation between the NAcc activation during winning and self-reports of pleasure from winning and relationship closeness. To this end, we executed a correlation analysis using the data from our NAcc ROI results (contrast OtherWin > NoWin) and the two behavioral measurement tasks (IOS and like winning for target). None of the six analyses proved significantly correlated after correcting for multiple comparisons (Bonferroni, *p* < .05). We next performed a whole-brain regression analysis to investigate the relation between neural activation patterns and self-reported behavioral data. This was done by implementing a repeated-measures analysis of covariance (ANCOVA) in SPM8 using the IOS scores as an additional regressor in the general linear model. This covariate analysis used the IOS scores as a covariate within the whole-brain BOLD response contrast for OtherWin. In other words, we examined whether self-reported measures for emotional closeness with a target can be associated with whole-brain activation when winning for that target (baselined against the NoWin contrast). The false-discovery-rate corrected (FDR cluster corrected) results did not show any activation clusters for any of the three targets.

## Discussion

The goal of this study was to test a new paradigm to measure neural correlates of vicarious joy. Vicarious joy is an aspect of empathy that was expected to be influenced by the emotional closeness to the target of the reward (Braams, Peters, Peper, Güroǧlu, & Crone, 2014b; Mobbs et al., 2009; Royzman & Rozin, 2006). In this study, we specifically investigated the neural processing of vicarious reward for, and its dependency on, a close (mother, father) versus a distant target (stranger). An additional focus was to compare vicarious joy responses towards fathers versus mothers, as fathers are unrepresented targets in prior research (Morelli et al., 2015). In line with earlier research, the NAcc responded to winning a reward for oneself, as well as when vicariously winning for close others (Braams & Crone, 2017; Morelli et al., 2015; Spaans, Burke, et al., 2018a). No such effect was found when winning for strangers. Interestingly, this study revealed different activation patterns when vicariously gaining for fathers compared with mothers, showing less activation in the NAcc when winning for self at the expense of fathers. Additional activation was observed in areas related to mentalizing and social moral processing when father wins (Moll, Zahn, de Oliveira-Souza, Krueger, & Grafman, 2005; Rilling, Sanfey, Aronson, Nystrom, & Cohen, 2004). The discussion is organized along the lines of these main findings.

This study employed a novel paradigm intended to investigate vicarious joy and empathy, which was based on prior research on vicariously gaining for charity (Spaans, Peters, et al., 2018b). Because of our focus on the family, we selected the mother, the father, and an unknown stranger as the baseline targets. This allowed us to develop a broader perspective on the emotional relationships the participants have with different targets. An important component of this task compared with prior research (Braams & Crone, 2017) was that outcomes for the participant and for the targets were presented at the same time, similar to a prisoner’s dilemma format (Doebeli & Hauert, 2005). This allowed us to put real-life costs on winning for someone else, making vicarious joy, in this design, arguably more prosocial. To validate the task manipulations, participants were asked to indicate how much they experienced joy when winning for self, parents, and strangers. As predicted, winning was experienced as most pleasurable for self and close others (parents) and less for strangers. There was a strong correlation found between IOS scores and like-winning results in father and stranger, but not for the mother. We believe this to be the case due to high heteroscedasticity and nonnormal distribution of the data at the high end of both scales (see Supplementary Material). These results set the stage for examining neural responses to winning for different targets.

Armed with this new paradigm we were able to replicate earlier findings. For one, when winning for self, compared with not winning, the participants showed increased activation in the NAcc. Thus, we were able to show personal reward processing in the ventral striatum when winning for self, replicating a long history of research (Berridge & Kringelbach, 2015; Delgado, 2007; Haber & Knutson, 2010; Knutson, Adams, et al., 2001a). This is also in line with earlier research on personal versus vicarious reward processing (Guassi Moreira & Telzer, 2018; Morelli et al., 2015). Region of interest analyses further revealed a similar NAcc pattern when generally contrasting outcomes for the parents with outcomes for the stranger. Similar results were found in previous studies that focused on the mother (Braams & Crone, 2017). This result might be interpreted as an emotional salience of playing for close others compared with an unknown stranger. Prior research confirmed stronger NAcc responses during donating in participants who show stronger family assistance ties (Telzer, Masten, Berkman, Lieberman, & Fuligni, 2010), suggesting that this neural response might signal closeness to targets.

The neural results align with self-reported measures of how much participants liked winning money for their parents compared with a stranger. Subjective measures of like winning were correlated with results from a closeness questionnaire (Le et al., 2007) for father and stranger. This, again, is in line with whole-brain activation in the NAcc for family members more than for strangers. Consistent with a large literature, NAcc was most active when individuals gained for self. This was observed in both the stranger condition (see meta-analysis by Morelli et al., 2015), as well as in the mother condition (Braams & Crone, 2017). However, winning for self, when the father loses, resulted in less activation in the NAcc, relative to the other targets, or relative to the conditions in which both self and father did not gain anything. These findings were not driven by individual differences in closeness, but rather seemed to be related to different neural signatures for fathers than mothers.

Besides the aforementioned confirmatory results, we also explored neural signatures of winning for others versus not winning for others. A whole-brain analysis revealed a robust pattern of activation in the mPFC and midline cortical areas (precuneus) when winning for fathers. In other words, these regions seemed to process vicarious rewards for fathers even in conditions where the participants themselves won nothing. These regions correspond to earlier findings (Schreuders, Klapwijk, Will, & Güroğlu, 2018; Spaans, Burke, et al., 2018a) during donation and vicarious reward tasks and are known to belong to a social brain network (Blakemore, 2008; Frith, 2007; Kanske, Böckler, Trautwein, & Singer, 2015). Interestingly, prior research by Mitchell, Macrae, and Banaji (2006) and subsequent meta-analyses (Denny, Kober, Wager, & Ochsner, 2012) revealed that the medial PFC and precuneus are important regions for differentiating between self and distant and close others. Together with the results for the NAcc showing that neural responses to self-wins are smaller when the father loses, these findings warrant further investigation of the role of both parent relations. Possibly, winning for father is associated with relatively stronger self–other comparisons, which would fit with the larger role of medial PFC and precuneus when gaining for fathers and the less pronounced NAcc when gaining only for self. These questions would further benefit from more detailed behavioral results and relationship measures. It is possible that implicit factors concerning monetary rewards play a role in experiencing vicarious rewards for fathers (Clarke, 2005). This will need to be compared with their views on their mothers and the different roles of the parents when dealing with financial topics.

An intriguing question for future research concerns the divergence in neural processing between fathers and mothers. The behavioral results for the IOS showed that even though emotional closeness was somewhat higher towards mothers than fathers, closeness was much lower for strangers. Therefore, it is unlikely that the effects are driven by differences in closeness to the target. Perhaps there are other variables that we have not measured that might play an influencing role. The father–child relationship might be more complicated than the IOS allows us to quantify. Potentially, fathers are still perceived differently than mothers in monetary contexts (Clarke, 2005). We did not, for example, collect the different roles of the parents when it comes to generating income for the family. In addition, prior studies have demonstrated gender-differentiated parenting styles towards aggressive behavior in young children, further suggesting that relationships may be influenced by for example gender-typical behaviors (Endendijk et al., 2017). Currently, this study underlines the strong need to focus on broader family relations rather than only the mother–child relationships and to extend our understanding of family context to mothers and fathers.

### Limitations

The current study has several potential limitations. First, the emotional closeness instrument used lacked the needed depth and granularity. The IOS has been shown to provide a reliable association to relationship closeness (Agnew, Loving, Le, & Goodfriend, 2004), as well as measures of frequency of contact, felt closeness, and behaviors associated with emotional closeness (Aron, Aron, & Smollan, 1992). But, in effect, it remains a short screening tool for something as complex as socioemotional relationships between children and their parents. A single item will likely lose some of the complex and nuanced differences in relationships participants have with their mother compared with their father. These differences would have helped connect our neural results to more meaningful behavioral differences in attitude and relationship to the three targets. Future studies need to provide a more faceted and complete measurement device to capture a fuller range of emotional closeness.

Second, the vicarious reward paradigm was a trade-off between design simplicity and allowing for player choices. The participants lacked volition and choices within the task, where outcomes independent of participants’ choices were presented. In future research, it will be valuable to combine the current paradigm with an active learning context and use prediction error model-based analyses (Burke, Tobler, Baddeley, & Schultz, 2010). The upside of this design, and the reason for it in the first place, was the exclusion of confounding neural processes as seen in previous donation tasks. Donation tasks are known for providing notoriously skewed results, with most people answering on one end of the scale, leading to heterogeneous response patterns and lack of power between the conditions. Within our paradigm, we were able to control for this, and as a result, we are able to assure high statistical power across all trials and conditions that were equally sampled. Future studies on vicarious joy would benefit from an associated behavioral task outside the scanner. This would allow for a more in-depth understanding of individual differences in behavior and its association with underlying personal neural processes. Finally, the amount of monetary outcome (1€ or 2€) was low for simplicity but a higher amount might provide higher activation amplitudes within the reward centers of the ventral striatum thereby increasing the coveted signal to noise ratio (Spaans, Peters, et al., 2018b). One other potential factor influencing the results could be based on reward magnitude. Perhaps the reward magnitude is processed differently depending on the outcomes of others. In a prior study in which magnitudes were varied to control for this effect, we found that NAcc responses were dependent on absolute magnitudes and did not interact with context (Spaans, Peters, et al., 2018b). However, this study focused on vicarious gains for charity, and it remains to be determined whether this is also the case for close family members. In future research, it will be interesting to examine relative gains for family members in more detail by varying not only outcomes but also magnitudes.

In addition, our sample size did not allow for an investigation into gender difference for vicarious reward processing and whether the gender combination between child and parent plays a role. Future studies should allow for gender as a covariate to elucidate potential differences. In addition, our current experimental design did not allow for real-life interaction between the participants and the targets of the false-choice fMRI task. Future research could aim to involve the parents as well as a stranger more directly during the scanning phase, to improve generalizability and ecological validity.

One final limitation relates to the relative homogeneity of our sample. Our sample consisted of young Dutch university students and should therefore be seen as a representation of that population. This homogeneity, of course, limits the degree to which these results are generalizable to broader and more diverse populations.

### Conclusion

The aim of this study was to provide and test a new paradigm for investigating vicarious joy among different social targets. For this purpose, our sample consisted of young adults with well-adjusted family backgrounds and relationships. This allowed for a more standardized baseline and first controlled experiment of our ideas and design. We were able to confirm ventral striatum activation when winning for oneself*,* and comparable results when gaining for mothers. In addition, a further differentiation on the whole-brain level was observed between the father and mother targets. Winning for fathers versus not winning for fathers exhibited a unique activation pattern in social and mentalizing areas (Mills, Lalonde, Clasen, Giedd, & Blakemore, 2014).

This paradigm shows potential value for further research into socioemotional relationships also for other targets as well as populations. The current study presents a first step by creating and testing a novel paradigm that is useful to investigate important fundamental questions on family relationships, in-group versus out-group thinking, vicarious joy, and empathy. To this end, the current manuscript serves a more fundamental purpose, with its primary goal to provide a basic understanding of the neural underpinnings of vicarious joy. The current findings provide a starting point for a deeper understanding of individual differences in family relationships, and for investigating research questions, such as those related to cultural differences, as well as in relation to more clinical samples with malfunctioning family relationships*.* Potentially revealing research could compare levels of vicarious joy among different cultures. Beyond the family, other possible social groups could be investigated. This could include peers or siblings for in-groups, but equally interesting different out-group targets, such as rivals in sports or the classroom. A further focus on anti-social populations, as well as families with a clinical history, might provide useful insights into the fundamental neural processing of empathy and vicarious joy within the brain.

#### Author note

This work was supported by an innovative ideas grant of the European Research Council (ERC CoG PROSOCIAL 681632 to E.A.C.). We confirm that this work is original and has not been published elsewhere, nor is it currently under consideration for publication elsewhere. The authors thank all participants for their contribution. We also thank Lisa Kool, Dorien Huijser, Anna van Steenbergen, and Cevdet Acarsoy for their help with the data collection. A special contribution is owed to Suzanne van deGroep, who started and carried this entire project with unique professionalism, thank you!

The statistical fMRI group maps have been made available (https://neurovault.org/collections/UNRMPFBJ/). The current study was not preregistered.

## Electronic supplementary material

ESM 1 (DOCX 491 kb)
