# Supplementary material for: I am happy for us: Neural processing of vicarious joy when winning for parents versus strangers
Source: Cogn Affect Behav Neurosci. 2020 Oct 14;20(6):1309–22. doi: 10.3758/s13415-020-00839-9 (PMC7716820; doi:10.3758/s13415-020-00839-9)
Supplement: Supplementary file 1 — (DOCX 491 kb) [file 13415_2020_839_MOESM1_ESM.docx]

**Supplementary Material**

These supplementary materials provide additional and more extensive information to go alongside the main manuscript. Detailed information regarding the whole brain results and ROI bilateralism checks have been added here to provide the reader with necessary but extensive further information.

*Figure S1: Inclusion of Other in Self*


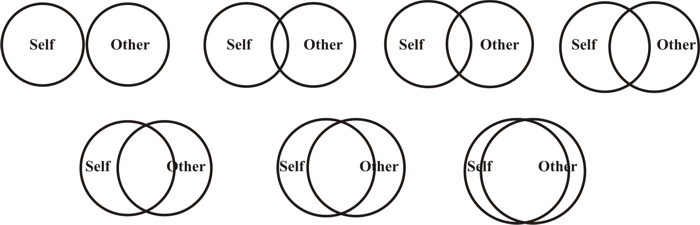


Figure S1:Inclusion of Other in Self. A one-item questionnaire on the perceived closeness between the participant and a specific ‘Other’. In our study, the targets of mother, father, and stranger were used to identify the ‘Other’.

*Figure S2: Behavioral Correlations Mother*


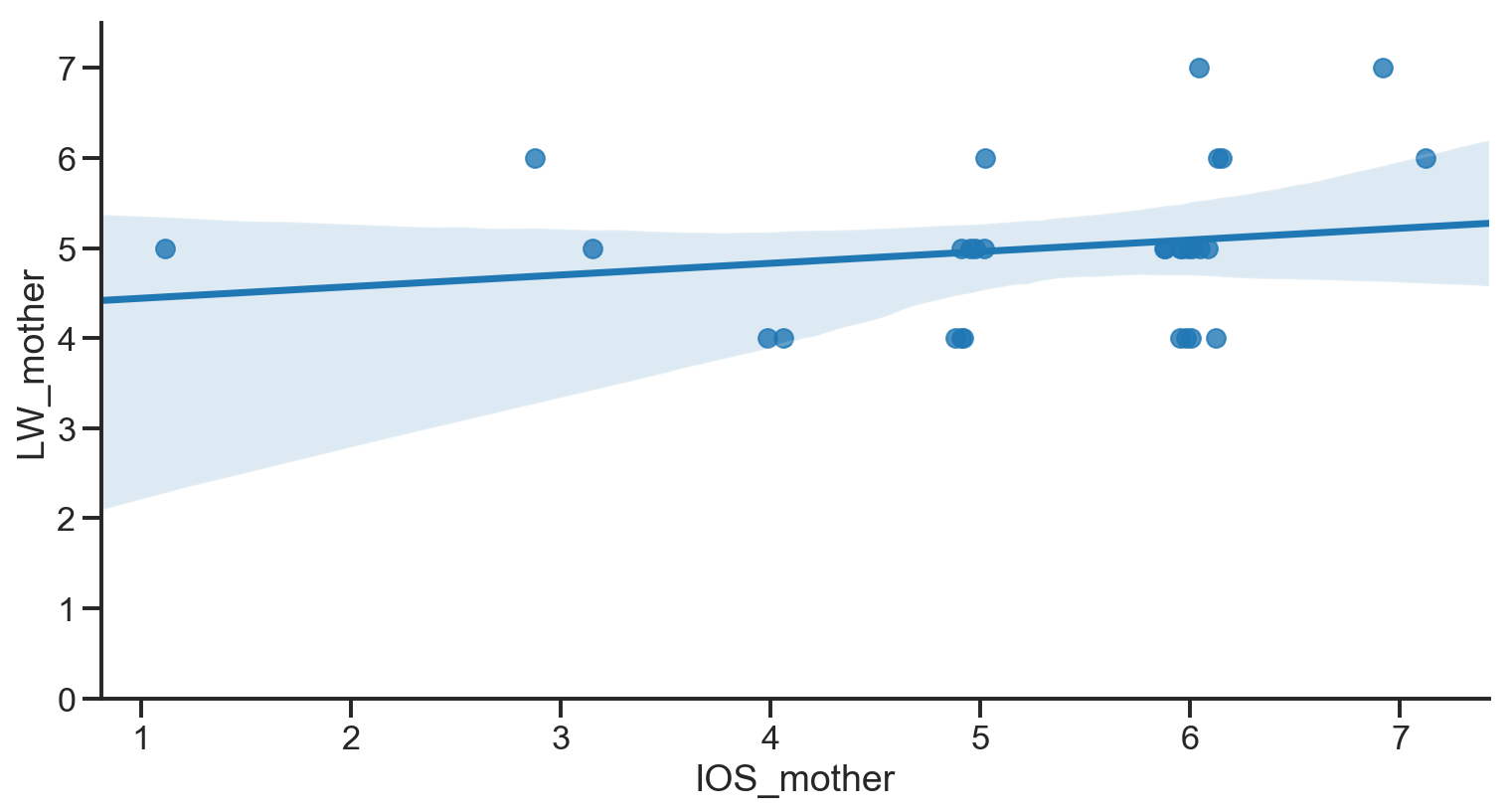


Figure S2: Scatterplot between Inclusion of Other in Self (IOS) and how much the participants liked winning for the Mother. Linear regression lines are plotted with a 95% confidence interval around it (blue shading). Due to many similar responses on the two Likert scales a 15% jitter was applied to the x axis to allow for better visibility of all data points.

*Figure S3: Whole brain all targets x conditions ANOVA*

*
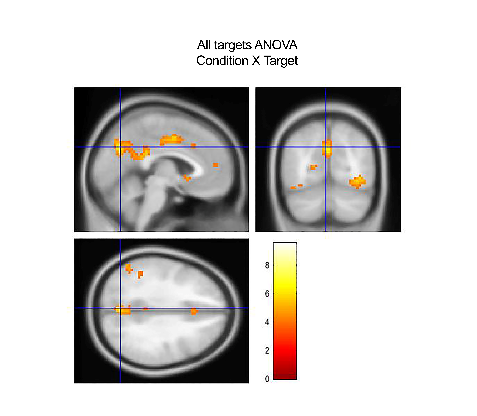
*

Figure S3: Whole brain results for a 3x4 ANOVA across all participants. Scattered activation pattern, with highest peaks found in the precuneus.

*Figure S4: NAcc hemisphere similarity check*


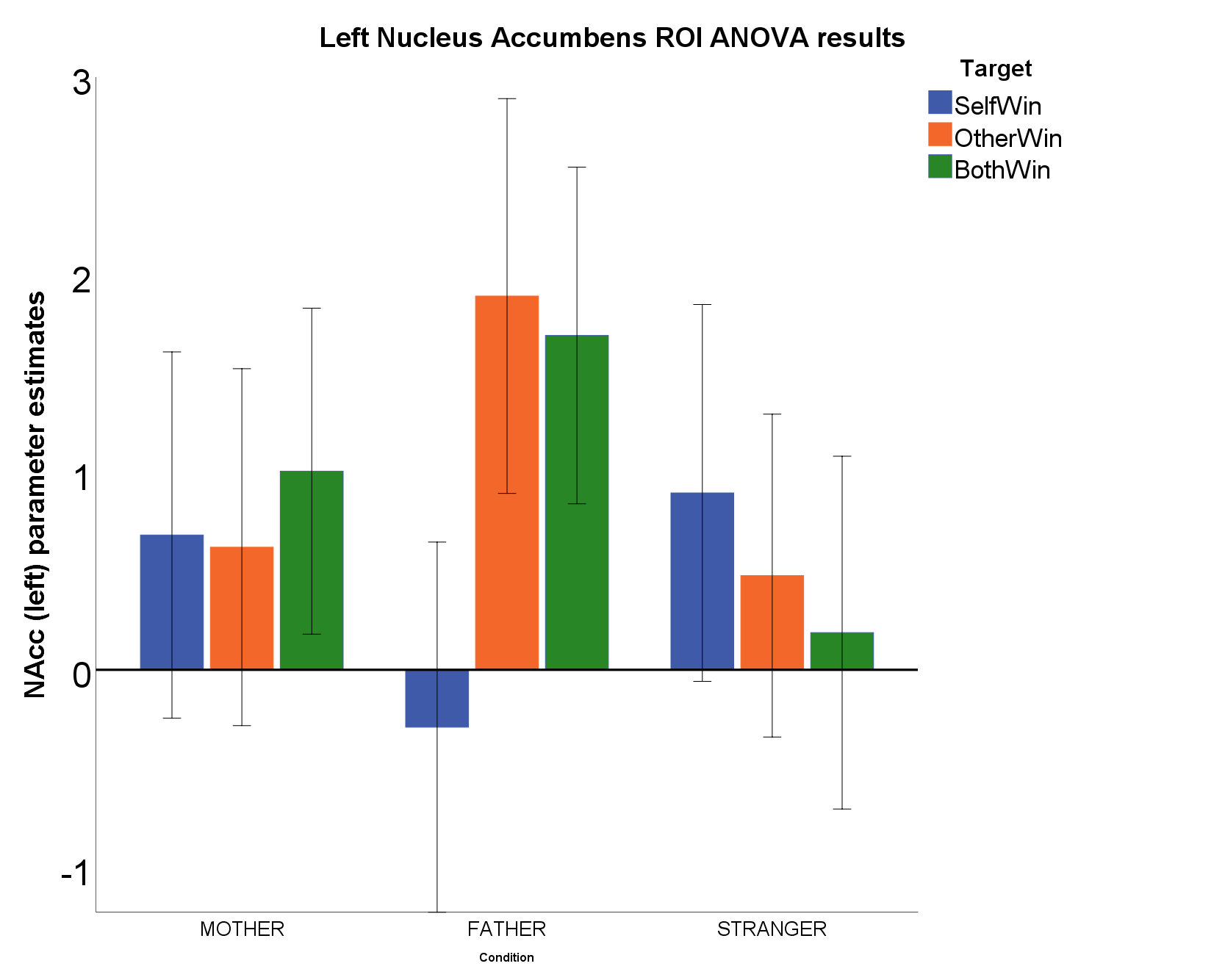

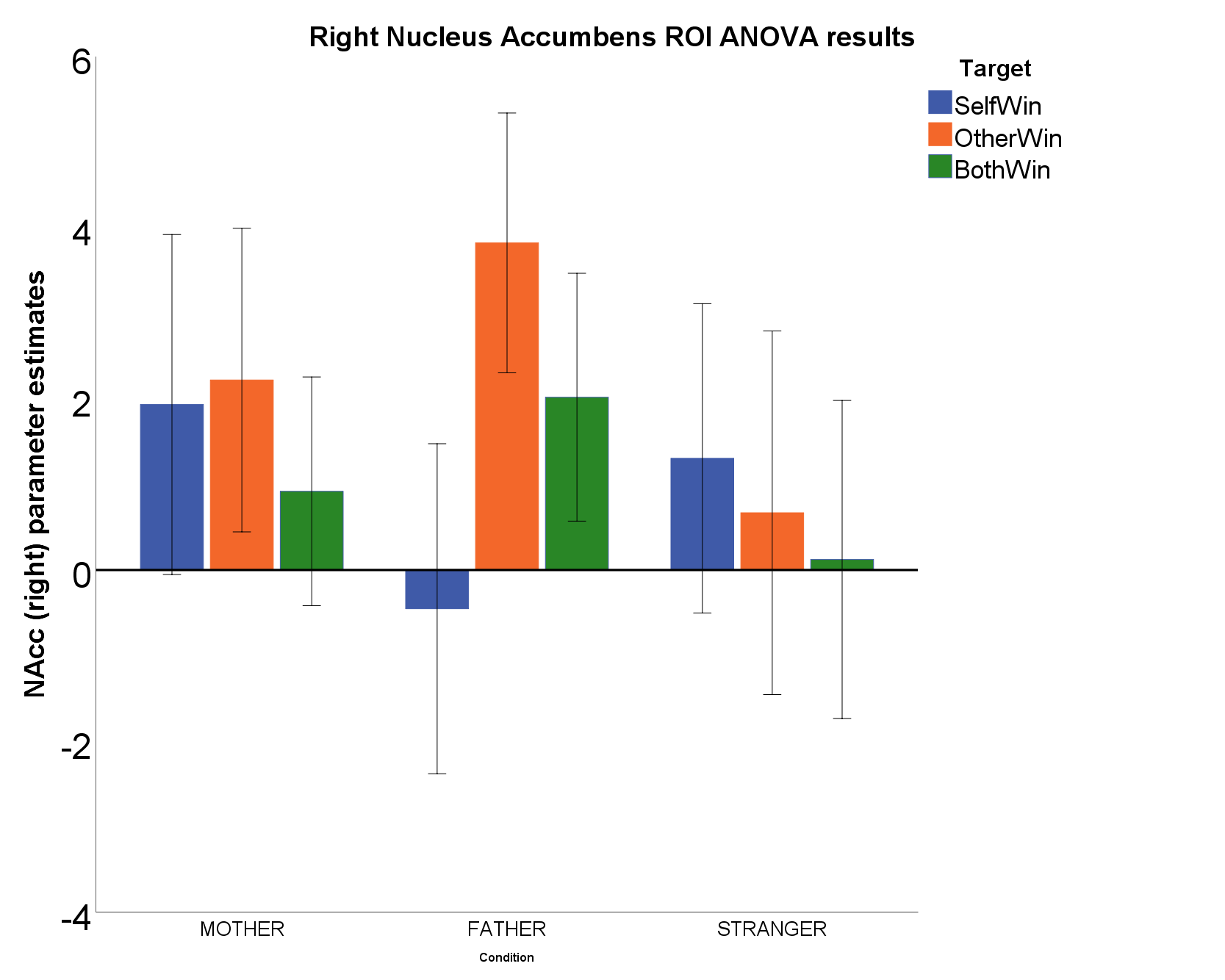


Figure S4: Nucleus Accumbens ROI results across all three targets for the three conditions (baselined against NoWin) for left and right NAcc separately. ROI. For Stranger the SelfWin condition shows the highest activation in both hemispheres. , For the mother condition we see similar activation patterns across the different outcomes in both hemispheres. Finally, for the father condition we see a clear divergence in the SelfWin condition in both hemispheres, which runs counter to the other two targets. Error bars represent 95% confidence interval of the mean

Figure S5: Time-series averages across three targets for SelfWin condition.


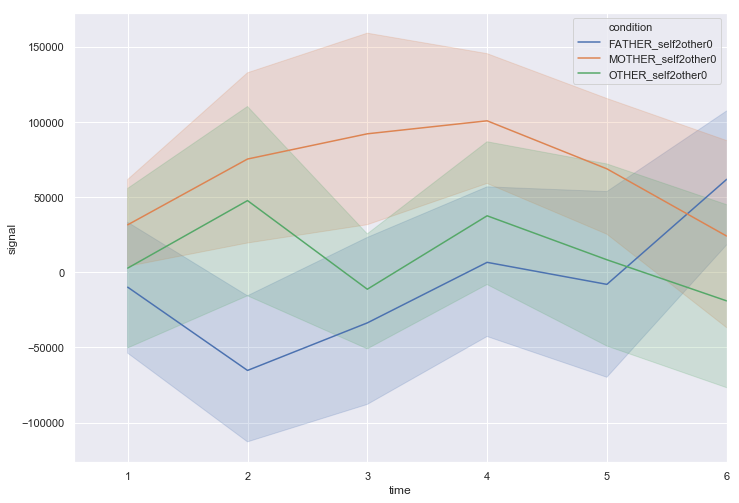


Figure S6: Group-level time-series results for all three targets (mother, father, stranger) from the moment of stimulus onset (first image of reward outcome) for SelfWin condition.
